# The Copper/Zinc Ratio Correlates With Markers of Disease Activity in Patients With Inflammatory Bowel Disease

**DOI:** 10.1093/crocol/otaa001

**Published:** 2020-01-23

**Authors:** Tobias Schneider, Daniel Caviezel, C Korcan Ayata, Caroline Kiss, Jan Hendrik Niess, Petr Hruz

**Affiliations:** 1 Department of Gastroenterology and Hepatology, Clarunis, Basel, Switzerland; 2 Department of Biomedicine, University of Basel, Basel, Switzerland; 3 Division of Clinical Nutrition, University Department of Geriatric Medicine Felix Platter, Basel, Switzerland

**Keywords:** inflammatory bowel disease, zinc, copper, copper/zinc ratio, trace elements, Crohn disease, ulcerative colitis

## Abstract

**Background:**

Zinc (Zn) and copper (Cu) are trace elements that serve as cofactors in catalytic processes with impact on immune responses. In patients with inflammatory bowel disease (IBD), decreased levels of serum Zn and Cu have been observed. Here, we investigated the effect of inflammation on serum concentrations of these trace elements in patients with IBD.

**Methods:**

In this cross-sectional study, 98 patients with Crohn disease (CD) and 56 with ulcerative colitis (UC) were prospectively enrolled. Disease activity parameters, such as C-reactive protein (CRP) and fecal calprotectin (FC) were compared to serum Zn, Cu, and Cu/Zn ratio.

**Results:**

Zinc insufficiency was observed in 11.2% of patients with CD and 14.3% with UC, Cu insufficiency in 20.4% with CD and 7.1% with UC. Anemia, hypoalbuminemia, increased FC, and elevated CRP were more frequently present in Zn-insufficient patients with IBD. In contrast, lower serum CRP values and a trend to lower FC were observed in Cu-insufficient patients. In multiple linear regression models adjusted for age, gender, and serum albumin, CRP positively correlated with serum Cu (*P* < 0.001) and the Cu/Zn ratio in both CD and UC (*P* < 0.001) but not with serum Zn concentrations. FC levels correlated only with the Cu/Zn ratio in patients with UC (*P* < 0.038).

**Conclusion:**

Systemic inflammation inversely affected the serum Zn and Cu concentrations and, consequently, resulted in an increased Cu/Zn ratio.

## Lay Summary

We found that zinc (Zn) and copper (Cu) insufficiency occurred in patients with inflammatory bowel disease and that systemic inflammation decreased serum Zn and increased Cu concentrations. This resulted in an increased Cu/Zn ratio. It may represent a novel parameter to assess disease activity.

## INTRODUCTION

Crohn disease (CD) and ulcerative colitis (UC) are chronic inflammatory conditions of the gastrointestinal tract often associated with malnutrition and weight loss caused by reduced intake, impaired absorption of nutrients, or a hypermetabolic state in patients with active disease.^1^ Patients with inflammatory bowel disease (IBD) can suffer from nutritional deficiencies of micronutrients (vitamins, minerals, and trace elements) and macronutrients (protein-energy malnutrition).^2^ Macronutrient deficiency has classically been associated with severe disease and has become less common in recent years with advances in treatment. However, the occurrence of micronutrient deficiencies especially of trace elements in IBD patients is underreported, and their consequences on disease progression and quality of life for the individual patient remains poorly understood.^2,3^

Zinc deficiencies are common in children and adults with IBD with a prevalence ranging from 15% to 40%.^4,5^ In contrast, serum Cu levels are reported to be higher or unchanged in patients with IBD compared to healthy controls.^6,7^ Possibly, a significantly lower Zn intake could contribute to Zn deficiencies observed in IBD patients as a cross-sectional analysis of dietary patterns of patients with CD revealed a significantly lower dietary intake of micronutrients, including Zn compared to healthy controls.^8^ In line with this observation, Zn intake reduces the risk of CD and has a protective effect on the development of UC.^9,10^ Zinc can reduce the duration and severity of diarrheal diseases.^11–13^ Mechanistically, Zn is a cofactor for many enzymes required for cell membrane repair, cell proliferation, growth, and immune system function.^14^ Zinc deficiency has been associated with the production of proinflammatory cytokines, including TNFα,^15,16^ disease protection in a murine colitis model,^17–19^ and increased oxidative stress.^20^ Copper serves as an essential cofactor for enzymes and electron transport proteins required for antioxidant, neurotransmitter, and histamine metabolism, oxidative phosphorylation, iron transport, and methionine and melanin synthesis.^3,21^ Thus, Cu deficiency results in anemia, peripheral and optic neuropathy, and myelopathy. In addition, animal studies and in vitro experiments indicate that Cu deficiency exerts numerous effects on the immune system by affecting the innate and adaptive immune response.^22^ For example, in biopsies of IBD patients in the inflamed mucosae, the content of the Cu-containing protein superoxide dismutase with antioxidant function was decreased, which may point to a diminished endogenous intestinal protection against oxygen-derived radicals in IBD.^23^

In acute infections, the 2 trace elements Zn and Cu react in an opposing manner to inflammatory stimuli.^24,25^ An elevated Cu/Zn ratio has been associated with malnutrition, increased oxidative stress, inflammation, and disrupted immune status in patients with chronic disease.^26^ However, how Zn and Cu concentrations correlate with inflammatory markers in IBD patients is yet to be understood. Here, we aimed to address the nutritional state of IBD patients by measuring the serum concentrations of trace elements Zn and Cu in IBD patients with quiescent and active disease.

## MATERIALS AND METHODS

### Participants and Study Design

In this cross-sectional study, patients with CD and UC were prospectively recruited between April 2016 and January 2017 at the Gastroenterology and Hepatology Department of the University Hospital Basel (Switzerland). Patients in our outpatient clinic and IBD patients hospitalized due to active exacerbated disease (with an evaluation within the first 3 days of hospitalization) were asked for participation. Out of 277 patients, 154 agreed to participate and, therefore, were included in this study. In all patients, the diagnosis of IBD was previously confirmed by endoscopy and histological analysis. Only 1 hospitalized patient could be included. On examination day, clinical data, procedures, and laboratory evaluation were assessed. Furthermore, dieticians assessed eating behavior and food supplements intake and performed a 24-hour food recall.

### Ethical Considerations

This study has been approved by the Ethics Committee of Northwest and Central Switzerland (EKNZ BASEC 2016-00071). Prior to inclusion, all patients gave their informed written consent.

### Blood and Fecal Samples

Patient blood samples were collected on the examination day in the hospital and analyzed for nutritional (trace elements, albumin) and inflammatory indices [C-reactive protein (CRP), leucocytes] according to our standard laboratory procedures. Serum Zn concentrations were determined by photometric analysis using the Wako Zinc test (Wako Chemicals, Neuss, Germany). Copper concentrations were measured with inductively coupled plasma mass spectrometry (ICP-MS) using iCAP Q ICP-MS (Thermo Scientific). Insufficiency of trace elements was defined as serum Zn <10.7 µmol/L; serum Cu <11 µmol/L in male and <12.6 µmol/L in female patients. To evaluate for intestinal inflammation, fecal calprotectin was assessed using a competitive chemiluminescent immunoassay (DiaSorin LIAISON Calprotectin, DiaSorin inc., Stillwater, MN, USA).

### Disease Activity

To evaluate the disease activity in patients with CD, the Harvey–Bradshaw Index (HBI) was assessed. The HBI is divided into 4 categories: remission (<5), mild disease,^5–7^ moderate disease,^8–16^ and severe disease (>16). For UC patients, the Modified Truelove and Witts Severity Index (MTWSI) were assessed: remission (≤ 3), mild disease,^4–6^ moderate disease,^7–11^ and severe disease (≥12). All scores were evaluated on the examination day.

### Data Analysis

Statistical analysis was performed using RStudio Team (RStudio: integrated Development for R. RStudio, Inc., Boston, MA, USA; Version: 1.1.442; 2016). Data were described as mean ± SD or as median and interquartile range (IQR) where appropriate. Differences in distribution for categorical parameters were calculated using chi-square test. To assess continuous data, students *t*-test and Mann–Whitney–Wilcoxon rank-sum test were used. The correlation between trace elements (Zn and Cu) and indices of inflammation (CRP and calprotectin) were investigated using multiple linear regression models for patients with UC and CD separately. Adjustment was performed for age, gender, and serum albumin as indicated. For the regression models, log-transformed values were used; back-transformed estimates represent ratios for categorical parameters and slopes for continuous variables with corresponding *P*-values. The level of significance was set as *P* < 0.05.

## RESULTS

### Baseline Characteristics and Serum Nutrient Deficiencies

In this study, 98 patients with CD and 56 patients with UC were analyzed. Demographic and patient characteristics are shown in Table 1. The mean duration of disease was 13.8 years for CD and 10.2 years for UC. The majority of patients were in remission (71.4% in CD, 55.4% in UC) as assessed by the clinical disease activity scores HBI and MTWSI. We found a fecal calprotectin (FC) of >100 µg/g stool in 61.2% in CD and 57.1% in UC samples. In Table 2, micronutrient results are displayed (Supplementary Table 1). Anemia was found in 29.4% of male and 25.5% of female patients for CD and in 12.0% of male and 12.9% of female patients UC. Hypalbuminemia was found in 32.7% in CD and 25% in UC patients.

**TABLE 1.** Demographic Characteristics in UC and CD Patients

	CD (n = 98)	UC (n = 56)
Age [years, mean (SD)]	41.32 (14.5)	41.6 (13.7)
Gender [male (n, %)]	50 (51)	25 (44.6)
Weight [kg, mean (SD)]	75.9 (20.3)	70.8 (16)
BMI [kg/m^2^, mean (SD)]	25.4 (6)	24.17 (4.3)
Smoker [n (%)]	27 (27.6)	11 (19.6)
Disease duration mean [years (SD)]	13.8 (10.8)	10.2 (9.4)
Montreal classification [n (%)]		
Age at diagnosis		
A1 (<16 years)	18 (18.4)	
A2 (16–40 years)	68 (69.4)	
A3 (>40 years)	12 (12.2)	
Disease location (UC)		
E1 (proctitis)		7 (12.5)
E2 (left colitis)		30 (53.6)
E3 (pancolitis)		19 (33.9)
Disease location (CD)		
L1 (terminal ileum)	19 (19.4)	
L2 (colon)	28 (28.5)	
L3 (ileum colon)	48 (49)	
L4 (upper GI)	1 (1)	
Others	2 (2)	
Disease behavior (CD)		
B1 (nonstenotic, nonpenetrating)	52 (53.1)	
B2 (stenotic)	23 (24.5)	
B3 (penetrating)	11 (11.2)	
B3p (perianal penetrating)	11 (11.2)	
Current use of medication for IBD [n (%)]		
5-Aminosalicylic acid	2 (2)	17 (30.4)
Immunomodulators	11 (11)	7 (12.5)
Integrin antagonists	6 (6.1)	11 (19.6)
TNFα blockers	69 (70.4)	24 (42.9
Glucocorticoids	13 (13.3)	8 (14.3)
Disease activity [n (%)]	HBI (CD)	MTWSI (UC)
Remission	70 (71.4)	31 (55.4)
Mild	13 (13.3)	15 (26.8)
Moderate	13 (14.3)	9 (16.1)
Severe	2 (2)	1 (1.8)
CRP >5 mg/l (n, %)	24 (24.5)	11 (19.6)
Calprotectin >100 µg/g (n, %)	60 (61.2)	32 (57.1)

HBI = Harvey–Bradshaw Index for CD (remission <5, mild 5–7, moderate 8–16, severe > 16); MTWSI = Modified Truelove Witts Severity Index for UC (remission ≤3, mild 4–6, moderate 7–11, severe ≥12).

**TABLE 2.** Prevalence of Insufficiencies in CD and UC patients

	CD (n = 98)	UC (n = 56)
Anemia Male (n, %)	15 (29.4)	3 (12)
Female (n, %)	12 (25.5)	4 (12.9)
Hypalbuminemia (n, %)	32 (32.7)	14 (25)
Zn insufficiency (n, %)	11 (11.2)	8 (14.3)
Cu insufficiency Male (n, %)	8 (15.7)	3 (12)
Female (n, %)	12 (25.5)	1 (3.2)

Anemia was defined when serum level was <140 g/L in male and <120 g/L in female patients. Hypalbuminemia: serum level <35 g/L. Trace elements insufficiency was defined: serum zinc <10.7 µmol/L; serum Cu <11 µmol/L in male and <12.6 µmol/L in female patients.

### Zinc Status and Correlation With Markers of Inflammation

In this study, insufficient serum Zn concentrations were observed in 11.2% of patients with CD and in 14.3% of patients with UC. Analysis of patients with IBD with insufficient Zn concentrations showed that they were more often anemic (52.6% vs 17.7%, *P* = 0.002) with significantly lower hemoglobin serum levels (128 g/L vs 140 g/L, *P* = 0.001) than patients with a normal Zn serum concentration (Table 3). The majority of patients with Zn insufficiency had hypalbuminemia, which occurred less frequently in the group with normal Zn concentration (63% vs 25%, *P* = 0.002). Moreover, Zn-insufficient patients with IBD showed significantly higher FC (560 µg/g vs 144 µg/g stool, *P* = 0.003) and serum CRP levels (3.8 mg/L vs 1.4 mg/L, *P* = 0.048). In the Zn-insufficient group, a nonsignificant trend toward lower body mass index (BMI) was observed. Other clinical parameters, such as smoking status, disease location, disease behavior, and the HBI score showed no significant differences between patients with Zn insufficiency and normal serum Zn concentration (data not shown).

**TABLE 3.** Analysis of Zn-Insufficient IBD Patients

All IBD Patients	Zn Insufficiency (n = 19)	Normal Zn (n = 135)	*P*-value
Gender [n (%)]			
Male	6 (32)	66 (49)	0.18
Female	13 (68)	69 (51)	(chi-q)
Diagnosis [n (%)]			
Crohn disease	11 (58)	87 (64)	0.76
Ulcerative colitis	8 (42)	48 (36)	(chi-q)
BMI [kg/m2, median (IQR)]	22.5 (21.3, 24.3)	23.7 (22.0, 27.3)	0.17
Inflammatory parameters [median (IQR)]			
CRP (mg/L)	3.8 (1.3, 8.0)	1.4 (0.7, 4.3)	**0.048**
Leucocytes (×10^9^/L)	7 (5.5, 8.2)	6.2 (4.9, 7.9)	0.378
Fecal calprotectin (µg/g stool)	560 (108, 1500)	144 (130, 149)	**0.003**
Hemoglobin [g/L, median (IQR)]	128 (118, 136)	140 (130, 149)	**0.001**
Anemia Male [n (%)]	4 (67)	14 (21)	**0.002**
Female [n (%)]	6 (46)	10 (14)	(chi-q)
Hypoalbuminemia [n (%)]	12 (63)	34 (25)	**0.002** (chi-q)

All *P*-values are calculated from unpaired *t*-test unless otherwise indicated. Anemia was defined when serum level was <140 g/L in male and <120 g/L in female patients. Hypalbuminemia: serum level <35 g/L.

Multiple linear regression analysis adjusted for age and gender indicated that serum Zn concentrations were inversely associated with FC levels and with serum CRP values in both CD and UC (Table 4). Clinical disease activity scores (HBI for CD and MTWSI for UC) correlated negatively with serum Zn concentrations in CD but not in UC (Table 5). In a subsequent multiple linear regression with additional adjustment for serum albumin levels, serum Zn concentrations were no longer significantly associated with CRP or FC levels (Table 6).

**TABLE 4.** Linear Regression of Log-Transformed Parameters and Inflammatory Markers in CD and UC Adjusted for Age and Gender

Parameter	Comparison	Group	Slope	*P*-value
Log serum Zn	Log CRP	CD	0.968	**0.009**
		UC	0.964	**0.039**
	Log calprotectin	CD	0.965	**0.002**
		UC	0.969	**0.019**
Log serum Cu	Log CRP	CD	1.140	**<0.001**
		UC	1.134	**<0.001**
	Log calprotectin	CD	1.042	**0.037**
		UC	1.052	**0.030**
Log Cu/Zn	Log CRP	CD	1.177	**<0.001**
		UC	1.177	**<0.001**
	Log calprotectin	CD	1.079	**<0.001**
		UC	1.086	**0.002**

Cu/Zn ratio: quotient of serum Cu to serum Zn; CRP: mg/L; calprotectin: µg/g stool.

**TABLE 5.** Linear Regression Between Trace Elements and HBI/MTWSI Scores Adjusted for Age and Gender

Parameter	Group (Score)	Slope/Ratio	*P*-value
Log serum Zn	CD (HBI)	0.987	**0.001**
	UC (MTWSI)	0.986	0.077
Log serum Cu	CD (HBI)	0.995	0.515
	UC (MTWSI)	1.012	0.343
Log Cu/Zn ratio	CD (HBI)	1.008	0.349
	UC (MTWSI)	1.026	0.098

Cu/Zn ratio: quotient of serum Cu to serum Zn; CRP: mg/L; calprotectin: µg/g stool.

**TABLE 6.** Multiple Linear Regression of Log-Transformed Parameters and Inflammatory Markers in CD and UC Adjusted for Age, Gender, and Serum Albumin

Parameter	Comparison	Group	Slope	*P*-value
Log serum Zn	Log CRP	CD	0.994	0.643
		UC	0.992	0.648
	Log calprotectin	CD	0.990	0.358
		UC	0.987	0.306
Log serum Cu	Log CRP	CD	1.152	**<0.001**
		UC	1.148	**<0.001**
	Log calprotectin	CD	1.030	0.165
		UC	1.043	0.082
Log Cu/Zn	Log CRP	CD	1.158	**<0.001**
		UC	1.156	**<0.001**
	Log calprotectin	CD	1.041	0.09
		UC	1.057	**0.038**

Cu/Zn ratio: quotient of serum Cu to serum Zn; CRP: mg/L; calprotectin: µg/g stool.

### Copper Status and Correlation With Markers of Inflammation

Next, we assessed Cu insufficiency in our IBD cohort. We found serum Cu insufficiency in 20.4% of patients with CD and in 7.1% with UC (Table 7). Patients with serum Cu insufficiency showed significantly lower serum CRP levels (0.4 vs 1.9 mg/L, *P* < 0.001) and lower BMI (21.9 vs 23.8 kg/m^2^, *P* = 0.02) compared to patients with normal serum Cu. No significant differences were found for age, gender, smoking status, disease location, and disease behavior.

**TABLE 7.** Analysis of Cu-Insufficient IBD Patients

All IBD Patients	Cu Insufficiency (n = 24)	Normal Cu (n = 130)	*P*-value
Gender [n (%)]			
Male	11 (46)	64 (49)	0.9
Female	13 (54)	66 (51)	(chi-q)
Diagnosis [n (%)]			
Crohn disease	20 (83)	78 (60)	0.05
Ulcerative colitis	4 (17)	52 (40)	(chi-q)
BMI [kg/m2, median (IQR)]	21.9 (20, 24.4)	23.8 (22, 27.1)	0.02
Inflammatory parameters [median (IQR)]			
CRP (mg/L)	0.4 (0.3, 0.7)	1.9 (1, 5.3)	**<0.001**
Leucocytes (×10^9^/L)	6.3 (4.7, 7.9)	6.2 (5, 7.9)	0.879
Fecal calprotectin (µg/g stool)	72 (41, 260)	188 (62, 559)	**0.092**
Hemoglobin [g/L, median (IQR)]	135 (128, 145)	139 (128, 148)	0.52
Anemia male [n (%)]	3 (27)	15 (23)	0.67
female [n (%)]	1 (8)	15 (23)	(chi-q)
Hypoalbuminemia [n (%)]	4 (17)	42 (32)	0.2 (chi-q)

All *P*-values are calculated from unpaired *t*-test unless otherwise indicated. Anemia was defined when serum level was <140 g/L in male and <120 g/L in female patients. Hypalbuminemia: serum level <35 g/L.

In a multiple linear regression analysis adjusted for age and gender, serum Cu correlated positively with CRP levels, as well as with FC in patients with CD and UC (Table 4). After additional adjustment for serum albumin, the correlation between serum Cu and CRP was still significant for UC and CD, whereas the correlation of serum Cu and FC was no longer significant (Table 6).

### Correlation Between the Cu/Zn Ratio and Markers of Disease Activity

**FIGURE 1.**
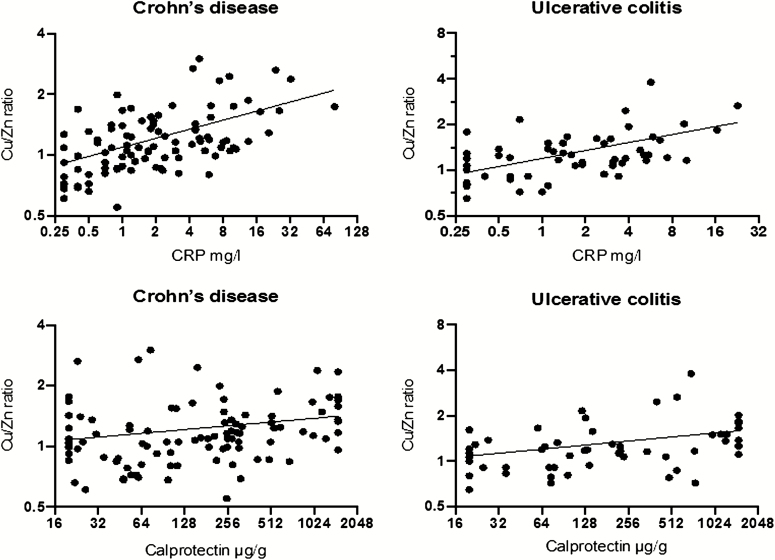
Linear regression adjusted for age and gender showed an association of the Cu/Zn ratio with CRP for CD and UC patients (both *P* < 0.001) and fecal calprotectin in CD (*P* < 0.001) and in UC patients (*P* = 0.002).

As an elevated Cu/Zn ratio has been previously associated with inflammation, we calculated the quotient of Cu and Zn (Cu/Zn ratio) and then compared to CRP values and FC stool levels in CD and UC patients (Fig. 1). The regression analysis with adjustment for age and gender (Table 4) revealed a significant, positive correlation between the Cu/Zn ratio and CRP and correlated significantly to FC in UC and equally in CD patients.

After additional adjustment for serum albumin, the correlation of Cu/Zn ratio and CRP was still significant for both UC and CD patients but, for FC, only in UC (Table 6). No significant correlations were observed between the Cu/Zn ratio and the HBI in CD and the MTWSI score in patients with UC (Table 5).

## DISCUSSION

Our cross-sectional analysis revealed that the serum concentrations of Cu and Zn are insufficient in a substantial proportion of patients with IBD. We observed increased serum Cu levels in patients with higher serum CRP values, while Zn levels decreased in patients with high CRP values and increased FC stool concentrations. Since these 2 trace elements respond in an opposite manner to inflammation, we investigated its quotient, the Cu/Zn ratio, and found a significant correlation of the Cu/Zn ratio with systemic inflammation in both CD and UC patients.

Zinc insufficiency occurred in up to 15% of our patients with IBD, while Cu insufficiency was present in up to 20% of our IBD patients. This phenomenon is of clinical importance because IBD patients with Zn deficiency have poor clinical outcomes indicated by increased hospitalization, surgeries, and complications rates in patients.^27^ Restoration of serum Zn values with Zn supplementation improved these disease outcomes.^27^ Mechanistically, an inappropriate antioxidant system could be involved as an association with disease activity in patients with IBD was shown for decreased serum Zn and for decreased superoxide dismutase activity, a Zn-containing enzyme with antioxidant activity.^28^ In addition, data from mouse models indicated that Zn deficiency aggravated colitis, induced the production of proinflammatory cytokine production, such as TNFα,^29,30^ and Zn supplementation has been shown to tighten the epithelial barrier.^31,32^ Our clinical data demonstrated that serum Zn levels correlated with clinical disease activity score HBI in patients with CD and with serum CRP concentration and fecal calprotectin levels. In line with these findings, studies investigating the relationship of the inflammatory response and circulating micronutrient concentrations report that Zn serum values can decrease by 10% during minor inflammation (CRP <15 mg/L) and by 40%–60% during major inflammation (CRP >100 mg/L).^25^

Because albumin is the main carrier protein of Zn in the serum, Zn concentrations will decrease when serum albumin is reduced. This can occur due to various reasons, such as low intake, malabsorption, or depletion during systemic inflammation in patients with active IBD. Thus, low serum albumin levels in patients with IBD has to be considered in the discussion of appropriate serum Zn concentrations. This is in line with a previous analysis of a nutritional screen cohort where Zn concentrations showed a positive association with serum albumin and a negative association with CRP.^33^ Systemic inflammatory response (as evidenced by elevated CRP) on the Zn concentrations could largely be adjusted to the albumin concentration by the calculation of a Zn/albumin ratio. However, whether this is important and measurements of Zn serum levels should be adjusted for serum albumin remains unclear as it was also observed that total enteral nutrition led to an increase in serum albumin and a decrease in disease activity but the serum Zn levels remained low.^34^ This suggests that, in some patients, Zn deficiency and hypoalbuminemia may occur simultaneously for independent reasons. Therefore, the actual prevalence of Zn deficiency in patients with IBD with active inflammation might be less prominent when patients with hypoalbuminemia are ruled out.

In contrast to Zn, Cu concentration rise during the acute-phase response.^35^ In the blood, coeruloplasmin binds approximately 95% of the Cu and, thereby, coeruloplamin and, as a consequence, Cu concentrations increase in the acute-phase response. Thus, in patients with active disease, Cu levels might be higher than in patients in remission.^36^ Copper levels were increased in patients with soft-tissue infections.^37^ This is in line with our observation of increased Cu levels in patients with high CRP and stool calprotectin. This may also explain the findings of epidemiological studies in mixed IBD populations where deficiency, normal, or even higher Cu levels compared to healthy controls were reported.^6,7,38^ The increase of serum Cu in patients with active inflammation could indicate that Cu deficiency may be underestimated in patients with active IBD.^25^

Because Zn and Cu respond in an opposite manner to inflammation, we considered that the Cu/Zn ratio could be a useful marker for active disease in IBD patients. Several lines of evidence indicated that the Cu/Zn ratio could indicate inflammation. For example, the Cu/Zn ratio was found to be elevated as acute-phase response in various infectious diseases, including in neonates giardiasis and amebiasis, as well as tuberculosis.^39–41^ Studies investigating the Cu/Zn ratio in cancer patients indicated an association of an increased Cu/Zn ratio in hepatocellular cancers, bladder cancer, and ovarian cancers.^42–44^ In IBD, the examination of the Cu/Zn ratio has been very limited so far. A higher Cu/Zn ratio has been described in patients with active colitis with elevated Cu and diminished Zn levels in serum of patients with IBD compared to healthy controls.^34^ These results support our findings that the Cu/Zn ratio correlated to CRP in CD and UC patients and to FC in patients with UC. Therefore, an increased Cu/Zn ratio may be observed in IBD patients with systemic inflammatory signs and may be a potential novel parameter of disease activity to be utilized in addition to CRP and FC levels in IBD patients.

A limitation in this study was the single-center, cross-sectional design with unequal group sizes and more patients in remission compared to active disease. Furthermore, there was no differentiation of patients in remission from deep remission foreseen as no endoscopic evaluation was performed at the time of evaluation. The individual assessment of dietary intake of micronutrients using a 24-hour recall strategy was of limited use as Cu and Zn are present in many food groups and several factors influence its absorption, the bioavailability, and, in consequence, the serum concentrations.

## CONCLUSION

In conclusion, we report here a significant correlation of the Cu/Zn ratio with CRP and calprotectin in patients with active IBD. If the Cu/Zn ratio is of diagnostic value for IBD patients needs to be addressed in future prospective studies by correlating the Cu/Zn ratio with endoscopic and histologic disease indices.

## Supplementary Material

otaa001_suppl_Supplementary_Table_1

## Declarations:

The study was approved by the Ethics Committee of Northwest and Central Switzerland (EKNZ BASEC 2016-00071).
