# Supplementary material for: The Copper/Zinc Ratio Correlates With Markers of Disease Activity in Patients With Inflammatory Bowel Disease
Source: Crohns Colitis 360. 2020 Jan 23;2(1):otaa001. doi: 10.1093/crocol/otaa001 (PMC7291944; doi:10.1093/crocol/otaa001)
Supplement: otaa001_suppl_Supplementary_Table_1 [file otaa001_suppl_supplementary_table_1.docx]

**Supplementary Table 1: Results of laboratory and nutritional markers**

| **Variable** | **CD (n = 98)** | | **UC (n = 56)** | |
| --- | --- | --- | --- | --- |
| **Hemoglobin (g/l) male**  **female**  **Thrombocytes (x10^9^/l)**  **Albumin (g/l)**  **Alkaline phosphatase (U/l)**  **Sodium (mmol/l)**  **Calcium (mmol/l)**  **Magnesium (mmol/l)**  **Potassium (mmol/l)**  **Selenium (µmol/l)**  **Zinc (µmol/l)**  **Copper (µmol/l)**  **Vitamin A (µmol/l)**  **Vitamin B12 (µmol/l)**  **Vitamin B6 (nmol/l)**  **Erythrocyte folate (nmol/l)**  **Triglyceride (mmol/l)**  **Cholesterin (mmol/l)**  **HDL cholesterin (mol/l)**  **LDL cholesterin (mmol/l)**  **Transferrin saturation (%)**  **Total iron binding capacity (µmol/l)**  **Soluble transferrin receptor (mg/l)**  **Homocysteine (µmol/l)** | | 147 (138, 155)  128 (120, 138)  256 (218.8, 302)  36 (34, 38.8)  60 (50, 72)  140 (138, 141)  2.3 (2.2, 2.3)  0.8 (0.8, 0.8)  3.9 (3.7, 4)  1.1 (0.9, 1.2)  12.9 (11.7, 14.3)  14.6 (12.4, 17.3)  1.6 (1.3, 2.1)  251 (181, 340)  119 (93.5, 165.5)  1517 (1160, 2463)  0.9 (0.6, 1.3)  4.2 (3.6, 4.9)  2.2 (1.8, 2.8)  1.5 (1.3, 1.7)  24 (18, 30)  66 (59, 71.8)  2.5 (2.2, 3.1)  13.1 (11.1, 17) | | 147 (144, 154)  134 (124, 137)  271 (224, 321)  37 (35, 39)  53 (46, 76)  140 (139, 141)  2.3 (2.2, 2.4)  0.8 (0.8, 0.8)  3.9 (3.7, 4)  1 (0.9, 1.2)  13.5 (11.9, 14.6)  15.3 (13.8, 18.3)  1.6 (1.4, 2)  287 (208, 375)  116 (92.8, 150.8)  1673 (1315, 2544)  0.9 (0.7, 1.2)  4.8 (4.3, 5.5)  2.6 (2, 3.3)  1.6 (1.3, 2)  25 (20, 32)  66 (59, 75)  2.8 (2.3, 3.3)  12.2 (9.4, 15) |

*All data are presented as median and interquartile ranges from blood serum analyses. CD: Crohn`s Disease; UC: Ulcerative Colitis*
